# Supplementary material for: The Wg and Dpp morphogens regulate gene expression by modulating the frequency of transcriptional bursts
Source: eLife. 2020 Jun 22;9:e56076. doi: 10.7554/eLife.56076 (PMC7340504; doi:10.7554/eLife.56076)
Supplement: Transparent reporting form [file elife-56076-transrepform.pdf]

eLIFE
1st Floor
24 Hills Road
Cambridge CB2 1JP, UK
P 01223 855340
W elifesciences.org
T @elife

eLife's transparent reporting form

We encourage authors to provide detailed information *within their submission* to facilitate the interpretation and replication of experiments. Authors can upload supporting documentation to indicate the use of appropriate reporting guidelines for health-related research (see EQUATOR Network), life science research (see the BioSharing Information Resource), or the ARRIVE guidelines for reporting work involving animal research. Where applicable, authors should refer to any relevant reporting standards documents in this form.

If you have any questions, please consult our Journal Policies and/or contact us: editorial@elifesciences.org.

Sample-size estimation

- You should state whether an appropriate sample size was computed when the study was being designed
- You should state the statistical method of sample size computation and any required assumptions
- If no explicit power analysis was used, you should describe how you decided what sample (replicate) size (number) to use

Please outline where this information can be found within the submission (e.g., sections or figure legends), or explain why this information doesn't apply to your submission:

Sample size was not computed when designing the study. Sample size for all experiments is listed under 'Data Analysis' in Methods. For Fano factor, average mRNA numbers, and for transcription site frequency and size, point-estimates and 95% confidence intervals were estimated by bootstrap technical replication with 10,000 bootstraps per estimate. For all such point-estimates, adequate sample size was confirmed by normal distribution of bootstrapped replicates.

Replicates

- You should report how often each experiment was performed
- You should include a definition of biological versus technical replication
- The data obtained should be provided and sufficient information should be provided to indicate the number of independent biological and/or technical replicates
- If you encountered any outliers, you should describe how these were handled
- Criteria for exclusion/inclusion of data should be clearly stated
- High-throughput sequence data should be uploaded before submission, with a private link for reviewers provided (these are available from both GEO and ArrayExpress)

Please outline where this information can be found within the submission (e.g., sections or figure legends), or explain why this information doesn't apply to your submission:

eLife Sciences Publications, Ltd is a limited liability non-profit non-stock corporation incorporated in the State of Delaware, USA, with company number 5030732, and is registered in the UK with company number FC030576 and branch number BR015634 at the address 1st Floor, 24 Hills Road, Cambridge CB2 1JP | August 2014

Replicate number for all experiments is listed under 'Data Analysis' in Methods. Cells with nuclei at least n = 100 pixels were carried forward for expression analysis. For single cell data, each segmented cell was considered a biological replicate. Since >1,700 cells were measured in one wing disc, each experiment utilized 3 wing disc replicates per genotype/gene. Thus, ~5,000 cells were individually measured per genotype/gene. Similar trends in RNA and transcription spots feature are observed in each disc individually, and hence, the analysis is not distorted by artifacts in pooling and cell segmentation. Source data is provided at https://doi.org/10.21985/n2-rfax-bk36.

Statistical reporting

- Statistical analysis methods should be described and justified
- Raw data should be presented in figures whenever informative to do so (typically when N per group is less than 10)
- For each experiment, you should identify the statistical tests used, exact values of N, definitions of center, methods of multiple test correction, and dispersion and precision measures (e.g., mean, median, SD, SEM, confidence intervals; and, for the major substantive results, a measure of effect size (e.g., Pearson's r, Cohen's d)
- Report exact p-values wherever possible alongside the summary statistics and 95% confidence intervals. These should be reported for all key questions and not only when the p-value is less than 0.05.

Please outline where this information can be found within the submission (e.g., sections or figure legends), or explain why this information doesn't apply to your submission:

Calculation of all point-estimates and 95% confidence intervals is described in the Methods section and/or relevant figure legends. For Fano factor, average mRNA numbers, and for transcription site frequency and size, point-estimates and 95% confidence intervals were estimated by bootstrap technical replication. For all such point-estimates, adequate sample size was confirmed by normal distribution of bootstrapped replicates. All data are plotted to show 95% confidence intervals of fit or point-estimate.

(For large datasets, or papers with a very large number of statistical tests, you may upload a single table file with tests, Ns, etc., with reference to sections in the manuscript.)

Group allocation

- Indicate how samples were allocated into experimental groups (in the case of clinical studies, please specify allocation to treatment method); if randomization was used, please also state if restricted randomization was applied
- Indicate if masking was used during group allocation, data collection and/or data analysis

Please outline where this information can be found within the submission (e.g., sections or figure legends), or explain why this information doesn't apply to your submission:

Groups were allocated according to genotype and gene probed by smFISH. Data were gathered in the same experimental run for the same gene. For image data, all samples were masked during data collection. Segmentation, image processing and expression analysis were carried out for all groups identically with a computational pipeline. Sample labels were masked in all steps.

Additional data files ("source data")

- We encourage you to upload relevant additional data files, such as numerical data that are represented as a graph in a figure, or as a summary table
- Where provided, these should be in the most useful format, and they can be uploaded as "Source data" files linked to a main figure or table
- Include model definition files including the full list of parameters used
- Include code used for data analysis (e.g., R, MatLab)
- Avoid stating that data files are "available upon request"

eLife Sciences Publications, Ltd is a limited liability non-profit non-stock corporation incorporated in the State of Delaware, USA, with company number 5030732, and is registered in the UK with company number FC030576 and branch number BR015634 at the address 1st Floor, 24 Hills Road, Cambridge CB2 1JP | August 2014

Please indicate the figures or tables for which source data files have been provided:

Experimental data is provided for all main Figures 1-6. MATLAB scripts for cell segmentation, model parameters, and model simulations are provided at https://github.com/bakkerra/smfish_pipeline. All source data is freely available at https://doi.org/10.21985/n2-rfax-bk36
